# Expression Patterns of Immune Genes Reveal Heterogeneous Subtypes of High-Risk Neuroblastoma

**DOI:** 10.3390/cancers12071739

**Published:** 2020-06-30

**Authors:** Zhenqiu Liu, Christa N. Grant, Lidan Sun, Barbara A. Miller, Vladimir S. Spiegelman, Hong-Gang Wang

**Affiliations:** 1 Department of Public Health Sciences, Pennsylvania State University College of Medicine, 500 University Drive, Hershey, PA 17033, USA; sld656@126.com; 2 Division of Pediatric Hematology and Oncology, Department of Pediatrics, Penn State College of Medicine, 500 University Drive, Hershey, PA 17033, USA; bmiller3@pennstatehealth.psu.edu (B.A.M.); vspiegelman@pennstatehealth.psu.edu (V.S.S.); hwang3@pennstatehealth.psu.edu (H.-G.W); 3 Division of Pediatric Surgery, Penn State College of Medicine, 500 University Drive, Hershey, PA 17033, USA; cgrant4@pennstatehealth.psu.edu

**Keywords:** ultra-high risk neuroblastoma, clinically distinct subtypes, immune-related gene signatures, prognostic markers, biclustering, TP53 signaling

## Abstract

High risk neuroblastoma (HR-NB) remains difficult to treat, and its overall survival (OS) is still below 50%. Although HR-NB is a heterogeneous disease, HR-NB patients are currently treated in a similar fashion. Through unsupervised biclustering, we further stratified HR-NB patients into two reproducible and clinically distinct subtypes, including an ultra-high risk neuroblastoma (UHR-NB) and high risk neuroblastoma (HR-NB). The UHR-NB subtype consistently had the worst OS in multiple independent cohorts (p<0.008). Out of 283 neuroblastoma-specific immune genes that were used for stratification, 39 of them were differentiated in UHR-NB, including four upregulated and 35 downregulated, as compared to HR-NB. The four UHR-NB upregulated genes (ADAM22, GAL, KLHL13 and TWIST1) were all upregulated in MYCN amplified neuroblastoma in 5 additional cohorts. TWIST1 and ADAM22 were also positively correlated with cancer stage, while GAL was an independent OS predictor in addition to MYCN and age. Furthermore, we identified 26 commonly upregulated and 311 downregulated genes in UHR-NB from all 4723 immune-related genes. While 43 KEGG pathways with molecular functions were enriched in the downregulated immune-related genes, only the P53 signaling pathway was enriched in the upregulated ones, which suggested that UHR-NB was a TP53 related subtype with reduced immune activities.

## 1. Introduction

Neuroblastoma is a pediatric tumor that is derived from progenitor cells of the sympathetic nervous system. It is the most common extracranial tumor of childhood, and accounts for 8% of all childhood cancer, but 15% of pediatric tumor related deaths. It is also the most common malignancy overall in the first year of life and approximately 40% of patients are younger than one year at diagnosis, and the median age at diagnosis is only 18 months [1]. Neuroblastoma is a complex heterogeneous disease. its clinical presentation is quite diverse, ranging from asymptomatic incidental tumors to widespread metastases with systemic manifestations. The clinical and biological heterogeneity leads to different clinical outcomes ranging from spontaneous regression to inexorable progression, metastasis, and death, despite intensive therapy [1]. In practice, Neuroblastoma is commonly classified as low, intermediate, or high risk with the criteria defined by the International Neuroblastoma Risk Group (INRG) project [2]. In general, patients with stage 4 disease and older than 18 months at diagnosis or with greater than stage 1 MYCN-amplified neuroblastoma are classified as high-risk neuroblastoma (HR-NB). Despite extensive research and progress in cancer biology over the last decades, improvements in the clinical outcome of neuroblastoma have mostly been achieved for low- and intermediate-risk patients. High-risk neuroblastoma (HR-NB) remains a difficult-to-treat tumor that has benefited relatively little from research advancements [3]. As a result, the five-year overall survival rate for HR-NB remains below 50%, while the overall survival rates for low- and intermediate-NB are over 90%. Standard treatments of chemotherapy, radiotherapy, and surgical methods demonstrate particularly low efficacy for HR-NB patients, and they have significant long and short-term toxicities. Biologically, HR-NB evades the control of the immune system and manifests high cell heterogeneity, which limits the efficacy of standard approaches. Therefore, there is an urgent need for identifying gene signatures that are associated with clinical outcomes and discovering molecular aberrations as a potential therapeutic target for HR-NB.

Immunotherapy represents a promising approach for HR-NB. One big advantage of immunotherapy is that it has less long-term and cumulative toxicity, which is particularly important for children. However, to date, only one immune-drug Unituxin (a chimeric GD2-specific antibody) has been used as a component of combination therapy for HR-NB, and the treatments with Unituxin are far from optimal due to the pronounced side effects that are associated with on-target/off-tumor toxicity [4]. Moreover, although HR-NB is a heterogeneous tumor with poor response to treatment, they are currently treated in a similar fashion without additional risk stratification. It is crucial to further stratify the HR-NB patients into subgroups, deeply understand the biological mechanisms of each subtype, build subtype-specific models, and search for corresponding immune-related genes and signaling pathways for personalized treatment of HR-NB patients. However, only limited studies have attempted this in HR-NB patient stratification [5,6].

With rapid technological advances, various types of omics data with different sizes, formats, and structures have been growing at an unprecedented rate in the public domain. The dimension and complexity of such big data provides opportunities for uncovering intrinsic subtypes that can discriminate subtle clinical outcomes that traditional approaches failed to do. Particularly, gene expression patterns have been applied to detect the intrinsic subtypes of different cancers [7,8,9]. The most successful story is breast cancer, which leads to thousands of publications and the discovery of subtypes, such as triple negative breast cancer [10,11,12]. Therefore, in this pilot study, we propose detecting HR-NB subtypes with the expression patterns of immune-related genes in multiple cohorts.

## 2. Results

### 2.1. Expression Patterns of NB-Specific Immune-Related Genes Stratify HR-NB Patients into Clinically Significant Subtypes

**Figure 1:**
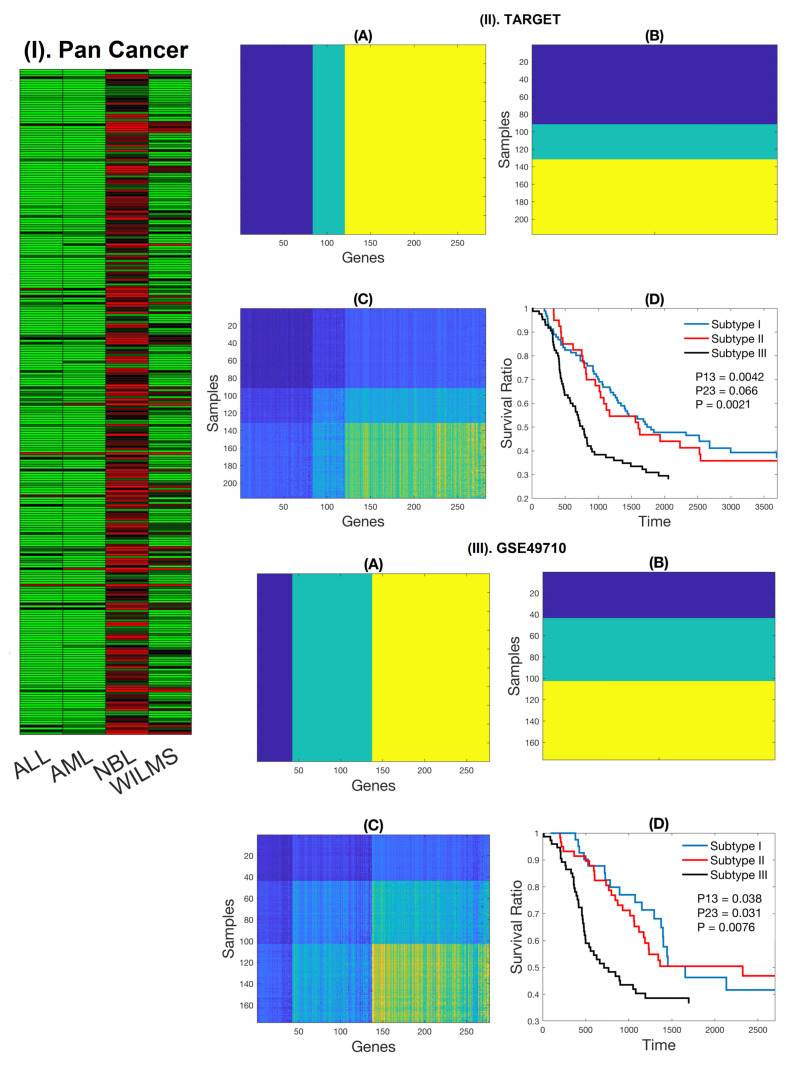
Co-clusters with 283 neuroblastoma (NB)-specific genes and their clinical relevance. (**I**) Heatmap with the average expression of 283 NB-specific immune-related genes in four pediatric tumors (data in Supplementary File S1). It shows that those genes are solely expressed in NB. (**II**), co-clustering with the 283 NB-specific genes of TARGET data. three clusters for both genes and samples are identified, leading to three clusters for patient samples. (**A**), visualization of clustering for genes. (**B**), visualization of clustering for samples. (**C**), visualization for both genes and samples. (**D**), Kaplan–Meier survival curve for three subtypes. (**III**), similar analysis with the 283 NB-specific immune gens in an independent cohort GSE49710. (**A**–**D**) are exactly the same analysis as (**II**).

With the RNA-seq data from pediatric pan-cancer, we compared the expression patterns of 4723 genes with immune functions in four pediatric tumors, including ALL, AML, NB, and WT with ANOVA and Student’s *t*-test, and identified 283 immune-related genes that were solely upregulated in NB. We defined those 283 genes as NB-specific immune-related genes. The NB-specific immune-related genes were chosen with more than two-fold higher in NB than any other three pediatric tumors and raw *p*-value $<1.0\times 10^{-5}$ without multiplicity correction as reported in Figure 1I, and Supplementary Table S1. The purpose of choosing NB-specific genes is two-fold. First, the expressions of those genes are easily detected in wet-lab validation. Secondly, we can reduce the number of candidate genes and computational complexity. Thus, they provided nice candidates for down-stream analysis and subtype-related studies.

Based on the 283 NB-specific immune-related genes, we applied the spectral co-clustering algorithm to two independent cohorts (TARGET and GSE49710) separately. There were a total of 217 and 176 HR-NB patients in TARGET and GSE49710, respectively. The algorithm identified three distinct clusters for both HR-NB samples and the 283 NB-specific immune genes in TARGET (Figure 1II and Supplementary Table S2). The Kaplan–Meier curve with three subtypes of samples for TARGET data was also plotted (Figure 1II(D)). Similar results were found in an independent GSE49710 cohort (Figure 1III), validating the findings of each other. Strikingly, we discovered that subtype III had the worst overall survival (OS) in both TARGET (*p* = 0.0021) and GSE49710 (*p* = 0.0076), when compared to subtypes I and II HR-NB samples together, while the survival differences between subtypes I and II were not statistically significant in either cohort. Therefore, the original HR-NB patients were stratified into two clinically distinct subgroups and we defined subtype III as the ultra-high risk neuroblastoma (UHR-NB), and subtypes I and II together as the new high risk neuroblastoma (HR-NB). We further compared the survival curves of UHR-NB with that of HR-NB subtype (Supplementary Figure S1) with the two cohorts. Amazingly, the results are remarkably consistent in two independent cohorts that were collected from different platforms. For the overall survival of TARGET data, patients in UHR-NB increased the hazard by 68% when comparing to patient in HR-NB subtype (HR: 1.68, 95% CI: 1.17–2.4. P: 0.0021) (Supplementary Figure S1A), while a similar hazard ratio was achieved with the GSE49710 cohort. Patients with UHR-NB subtype of the later cohort increased the hazard by 73% comparing to its HR-NB subtype (HR: 1.73, 95% CI: 1.12–2.67, P: 0.00758) (Supplementary Figure S1B). More impressively, the two-year survival rates of UHR-NB subtype are 48.8% and 43.2% for TARGET and GSE49710, respectively, while the corresponding two-year survival rates for HR-NB subtype are 79% and 75%, indicating substantial survival difference in both cohorts. More than half of the UHR-NB patients were deceased within two years, when comparing to less than one-fourth of the subjects in HR-NB.

**Figure 2:**
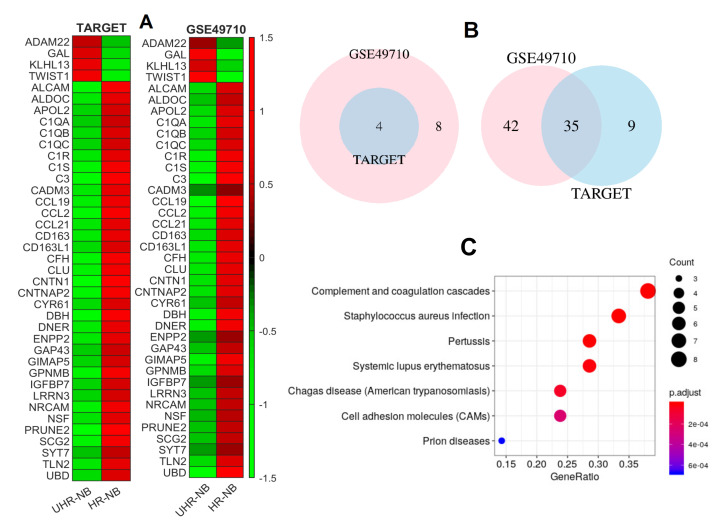
The 39 commonly differentiated NB-specific immune genes and their biological characterization. (**A**) Heatmaps of the 39 commonly differentiated immune-related genes in both cohorts. (**B**), Venn diagram depicting the number of commonly differentiated NB-specific immune-related genes, where the left and right diagrams were for the up- and down-regulated genes, respectively, and (**C**), enriched KEGG pathways for 35 commonly down-expressed NB-specific immune-related genes in ultra-high risk neuroblastoma (UHR-NB).

The heatmaps for the 39 commonly differentiated immune-related genes (Figure 2) in both cohorts showed that only four NB-specific immune genes were upregulated, while 35 of them were downregulated, indicating that inflammatory and immune cytolytic activities were depressed in UHR-NB. The Venn diagrams (Figure 2B) demonstrated the overlaps of both upregulated and downregulated immune-related genes in two independent cohorts. We used a Jaccard index J=|A∩B|/(|A|+|B|−|A∩B|) to measure the overlapping, where |.| represented the number of elements in a set. The Jaccard index for upregulated genes was 50% (left, Figure 2B), and was 40.7% for down-expressed genes (Right, Figure 2B), distinct from random subset intersections. Enrichment analysis with 35 downregulated NB-specific immune-related genes demonstrated that those genes were enriched with seven KEGG pathways (Figure 2C) with such a small number of genes. These pathways included Complement and coagulation cascades, *Staphylococcus aureus* infection, *Pertussis*, Cell adhesion molecules(CAMs), and others. Particularly, the top KEGG pathway, Complement and coagulation cascades, was an interesting one. It was a component of innate immunity and an ancient defense mechanism against invading pathogens. It was also considered as an effector arm of the immune system that contributed to the destruction of tumor cells. Many therapeutic strategies were proposed based on the intensification of complement-mediated responses against different tumors over the decades. However, the functions of the Complement and coagulation cascades pathway was complex. It might also support chronic inflammation, promote an immunosuppressive microenvironment, induce angiogenesis, and activate cancer-related signaling pathways [13]. As a result, the complement and coagulation cascades pathway might also enhance tumor growth and increase metastasis [14]. In our analysis, the complement and coagulation cascades pathway was enriched in HR-NB and it had lower activities in UHR-NB, which suggested important functional mechanisms in UHR-NB.

### 2.2. Thirty-Nine NB-Specific Differentiated Immune-Related Genes and Their Biological Characterization in UHR-NB and HR-NB Subtypes

With the 283 NB-specific immune-related genes, we next performed a *t*-test to identify 39 commonly differentiated NB-specific immune-related genes for UHR-NB vs HR-NB subtype. The genes were identified with the criteria of more than 1.5-fold changes and the raw cutoff *p*-value of $1\times 10^{-3}$ without multiplicity correction. The differentiated immune genes in each cohort (TARGET and GSE49710) and their fold changes and *p*-values were reported in Supplementary Table S3. The heatmap and gene enrichment analysis were reported in Figure 2.

### 2.3. Validating Four Upregulated Immune-Related Genes in UHR-NB with Additional Independent Cohorts

Four NB-specific immune-related genes upregulated in UHR-NB included ADAM22, GAL, KLHL13, and TWIST1. ADAM22 is a member of the ADAM (a disintegrin and metalloprotease domain) family. It is a cell surface protein in a disintegrin domain. ADAM22 is known to function in cellular migration and differentiation, and it is involved in many biological processes that are related to cell-cell and cell-matrix interactions. These processes may be critical to the metastatic phenotypes [15]. GAL (GAL1) is a glycan-binding protein and a member of prototypical group. It is a multifunctional protein that is involved in development and differentiation. There is some evidence that GAL plays a critical role in immune responses and angiogenic processes [16]. On the other hand, KLHL13 (Kelch Like Family Member 13) belongs to KLHL family and its role in disease is less studied in the literature. Finally, TWIST1(twist family bHLH transcription factor 1) is a highly conserved transcription factor that belongs to the family of basic helix–loop–helix (bHLH) proteins. It plays an important role in differentiation and the inhibition of apoptosis. There is some evidence that TWIST1 promotes the metastatic dissemination of cancer cells and induces chromosomal instability [17]. We intended to explore the associations between known NB markers and the four UHR-NB genes.

**Figure 3:**
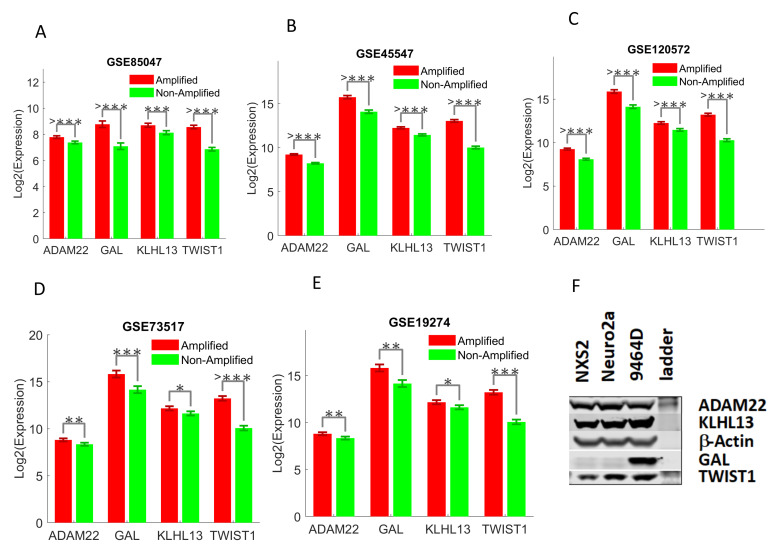
Four UHR-NB associated genes in MYCN amplified and non-amplified Tumors in five additional cohorts and mouse cell lines. The error bar denoted the mean expressions plus and minus one standard deviation. The raw *p* -values from student’s *t* test were reported on (**A**–**E**): *: *p* = 0.05–0.01, **: *p* = 0.01–0.001, ***: *p* = 0.001–0.0001, and >***: *p* < 0.0001. (**F**): mouse cell lines, where NXS2 and Neuro2a are MYCN non-amplified cell lines and 9464D is a MYCN amplified cell line.

UHR-NB associated immune-related genes are upregulated in MYCN amplified NB. MYCN gene is a member of the MYC family of proto-oncogenic transcription factors. MYCN encodes a protein with a basic helix-loop-helix (bHLH) domain. It is a well-known risk factor of neuroblastoma, and MYCN amplification is associated with poor diagnosis and survival [18]. Two-sided student’s *t* test was used to detect the expression differences between MYCN amplified and non-amplified patient samples with five additional cohorts. The five cohorts include GSE85047 (55 amplified/222 non-amplified), GSE45547 (93 amplified/550 non-amplified), GSE120572 (83 amplified/310 non-amplified), GSE73517 (33 amplified/72 non-amplified), and GSE19274 (22 amplified/78 non-amplified). The four UHR-NB associated genes were consistently upregulated in MYCN amplified NB in all five cohorts (Figure 3A–E), indicating that UHR-NB was associated with MYCN amplified NB.

Particularly, Genes GAL and TWIST1 were strongly associated with MYCN amplification (*p* < 0.0001) in four or five independent cohorts. Interestingly, our findings with TWIST1 were conformed by two previous studies in vivo and vitro, showing that TWIST1 co-occupied enhancers with MYCN and was required for the expression of the MYCN enhancer axis [19], and it was a direct transcriptional target of MYCN [17]. We further validated the four genes with the mouse cell lines (Figure 3F). The immune-related genes ADAM22, KLHL13, and TWIST1 were expressed in both MYCN amplified and non-amplified cell lines, while GAL was significantly upregulated in MYCN amplified cell line (9464D) as compared to the non-amplified cell lines (NXS2 and Neuro2A).

**Figure 4:**
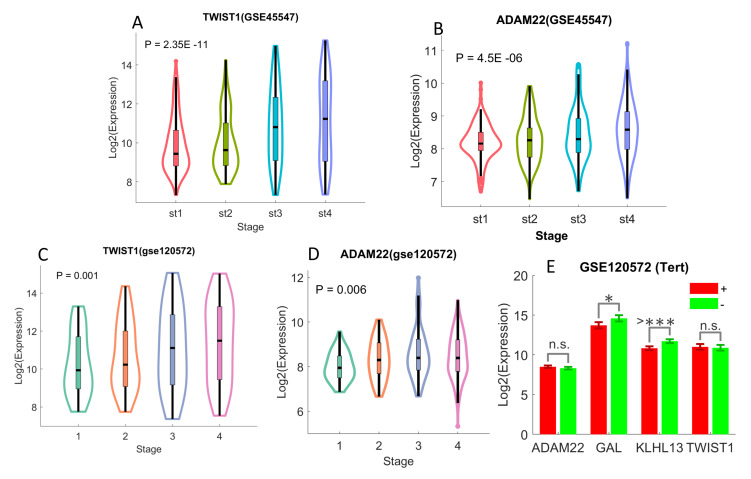
The associations of UHR-NB genes with tumor stage and TERT rearrangement. The error bar in E denoted the mean expressions plus and minus one standard deviation. The raw *p*-values reported in (**A**–**D**) were estimated with the one-way ANOVA, while the *p*-values in E were calculated with the student’s *t* test. (**A**) TWIST1 and stage in GSE45547. (**B**) ADAM22 and tumor stage in GSE45547. (**C**) TWIST1 and tumor stage in GSE120572. (**D**) ADAM22 and stage in GSE120572. (**E**) UHR-NB genes and TERT.

UHR-NB upregulated genes are associated with tumor-stage and telomere maintenance. We compared the expression levels of the four UHR-NB overexpressed genes to tumor-stage. One-way ANOVA was used to detect the expression differences in different tumor stages. Both TWIST1 and ADAM22 were upregulated for patients with higher tumor-stages in two independent cohorts, including GSE45547 and GSE120572 (Figure 4A–D).

TWIST1 had a stronger association with tumor stage, as it had relatively smaller *p*-values than ADAM22 in both cohorts. For instance, TWIST1 has the *p*-value of 2.35 × 10${}^{-11}$ in GSE45547 (Figure 4A), compared to the *p*-values of 4.5 × 10${}^{-6}$ in ADAM22 with the same cohort (Figure 4B). With a two-sided student’s *t* test, we also discovered that KLHL13 was highly differentiated (*p* < 0.0001) with different telomere maintenance statuses (Figure 4E). Telomere maintenances were related to TERT and MYCN alterations. Both TERT rearrangements and over-expression led to positive telomere maintenance. Positive telomere maintenance was essential for cancer cell to become immortal and it was related to poor prognosis [6]. KLHL13 was significantly upregulated in patients lacking of telomere maintenance. It might have tumor-protective effect.

**Figure 5:**
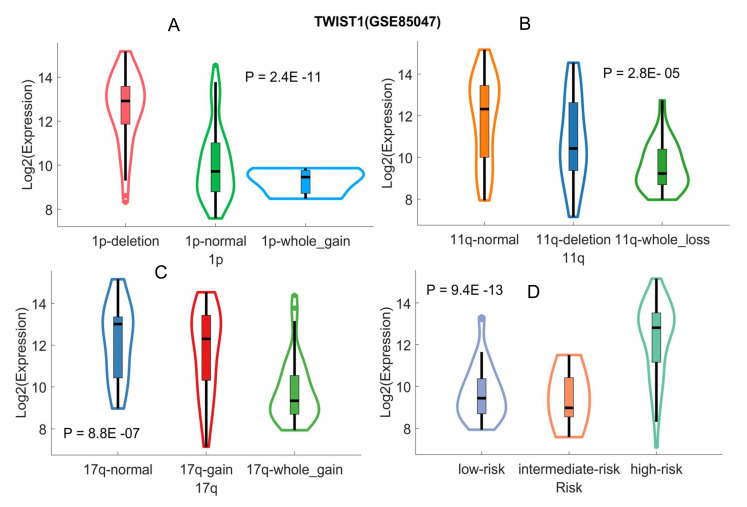
The associations of TWIST1 with genetic alterations and risk status. The *p*-values were estimated with the one-way ANOVA. (**A**) TWIST1 and 1p deletion. (**B**) TWIST1 and 11q deletion. (**C**) TWIST1 and 17q gain. (**D**) TWIST and risk status.

TWIST1 is strongly associated with 1p and 11q deletion, 17q gain, and risk status. Heterozygous deletions of 1p or 11q and gain of 17q are well-known genetic alterations and risk factors in HR-NB [20]. We further examined the associations between UHR-NB gene expressions and genetic alternations. One-way ANOVA was used to test the expression differences among different genetic alterations. Figure 5 reports the results.

Interestingly, there were strong linear correlations between TWIST1 expression and genetic alterations. TWIST1 was upregulated in patients with 1p deletion (Figure 5A, *p* = 2.4 $\times 10^{-11}$), downregulated in patients with 11q deletion (Figure 5B, *p* = 2.8 $\times 10^{-5}$), and downregulated in 17q gain (Figure 5C, *p* = 8.8 $\times 10^{-7}$). It was also upregulated in original HR-NB (Figure 5D, *p* = 9.4 $\times 10^{-13}$), indicating the importance of TWIST1 in HR-NB. The associations between other UHR-NB genes and genetic alterations were relatively weak.

The prognostic implications of UHR-NB upregulated genes.We performed Cox proportional hazard analysis on the original HR-NB samples of TARGET and GSE49710 cohort to further investigate the prognostic relevances of UHR-NB upregulated genes. Among four UHR-NB upregulated genes, GAL and TWIST1 were statistically significant predictors of overall survival for HR-NB in both cohorts without adjusting other known risk factors (Supplementary Table S4). The estimated parameter of GAL for TARGET was β=0.696 with the hazard ratio of 2.005 (95% CI, 1.43–2.81) and *p* value of 5 $\times 10^{-5}$, while the estimated coefficient of GAL for GSE49710 cohort was β=0.862 with the hazard ratio of 2.37 (95% CI: 1.53–3.66) and *p* value of 1 $\times 10^{-4}$. Similarly, the estimated coefficient of TWIST1 for TARGET was β=0.613 with hazard ratio of 1.85 (95% CI: 1.30–2.62) and *p* value of 6 $\times 10^{-4}$, while the estimated parameter of TWIST1 for GSE49710 was β=0.594 with the hazard ratio of 1.81 (95% CI: 1.11–2.95) and *p*-value of 0.017. After adjusting for other known risk factors including age and MYCN amplification, GAL was still highly significant in both of the cohorts. The estimated coefficient of GAL for TARGET was β=0.258 with the marginal hazard ratio of 1.29 (95% CI: 1.10–1.52) and the *p*-value of 0.0016, while the estimated parameter for GSE19710 was β=0.650 with the hazard ratio of 1.91 (95% CI: 1.18–3.10) and *p* value of 0.008 (Supplementary Table S4), indicating that GAL was an independent predictor of overall survival (OS) for HR-NB patients in addition to MYCN amplification and age. On the other hand, TWIST1 was only statistically significant in the TARGET cohort after adjusting for age and MYCN amplification. Its estimated parameter in TARGET was β=0.247 with the hazard ratio of 1.28 (95% CI: 1.06–1.54) and *p*-value of 0.0086, demonstrating that TWIST1 was an independent OS predictor in addition to age and MYCN amplification in one cohort.

### 2.4. Differentiated Immune-Related Genes in UHR-NB Subtype and Their Biological Significance

**Figure 6:**
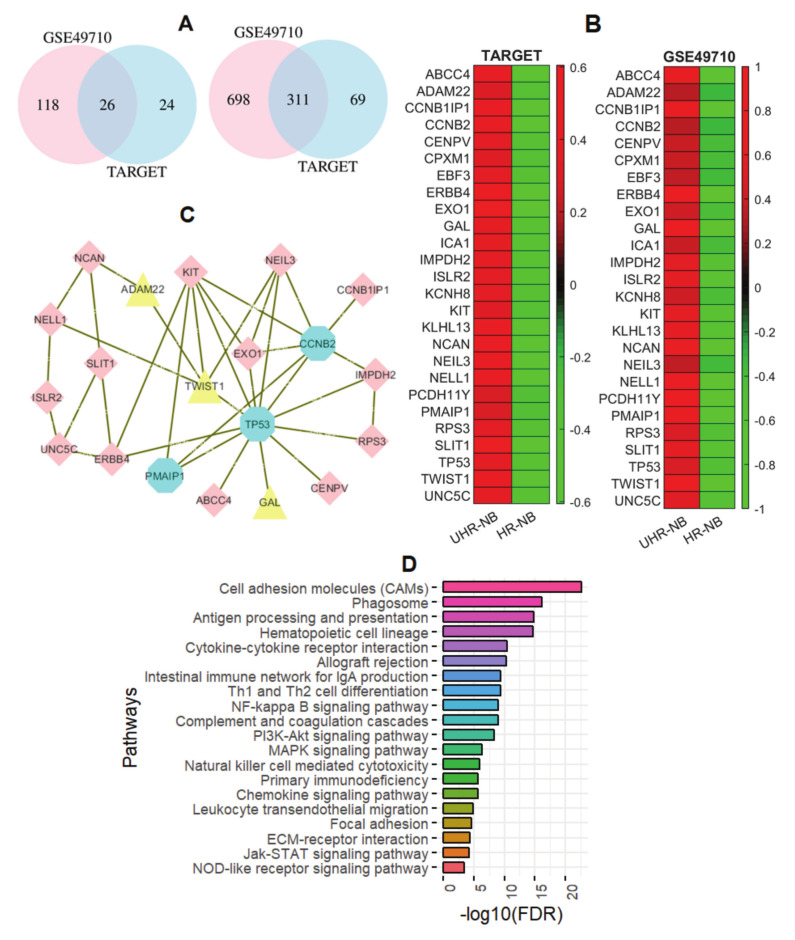
Enrichment analysis for differentiated immune-related genes in UHR-NB. (**A**) Venn diagrams for up- (left) and down- (right) regulated genes. (**B**) Heatmap for 26 upregulated genes. (**C**) Protein–protein interaction (PPI) network for 26 upregulated genes. (**D**) Top 20 immune-related pathways for 311 downregulated genes in UHR-NB, as compared to HR-NB subtype with STRING.

In order to further investigate the biological characterization of the UHR-NB subtype, we compared the expressions of 4723 immune-related genes downloaded from InnateDB (www.innatedb.com/) with TARGET and GSE49710, and identified 26 commonly upregulated immune-related genes (left panel of Figure 6A) and 311 commonly downregulated immune-related genes (right panel of Figure 6A) in UHR-NB in both cohorts (>1.5 fold, and $p<1.0\times 10^{-4}$).

The selected genes in each cohort were listed in Supplementary Table S5. The Jaccard indexes for upregulated and downregulated genes were 15.5% and 28.9%, respectively, which were significantly higher than random selection. Assuming that there were $n_{1}$ and $n_{2}$ genes in set 1 and set 2, respectively, and the probability to have *p* common genes was $1/\{{(}_{p}^{n_{1}}){(}_{p}^{n_{2}})\}$, which was very small.

UHR-NB subtype is associated with depressed expression of immune-related genes and Lower activities of immune-related pathways.There were many more (311) downregulated immune-related genes than (26) upregulated immune-related genes in UHR-NB, as compared with HR-NB, indicating reduced immune-related molecular functions in UHR-NB subtype. GSEA analysis with STRING demonstrated that the set of downregulated immune-related genes in UHR-NB were significantly enriched with 43 immune-related KEGG pathways with molecular, metabolic, and signaling functions (Supplementary Table S6). The top 20 immune-related pathways (Figure 6D) included Cell adhesion molecules (CAMs), Phagosome, Antigen processing and presentation, Hematopoietic cell lineage, Cytokine-cytokine receptor interaction, Allograft rejection, Intestinal immune network for IgA production, Th1 and Th2 cell differentiation, NF-kappa B signaling pathway, Complement and coagulation cascades, PI3K-Akt signaling pathway, MAPK signaling pathway, Natural killer cell mediated cytotoxicity, primary immunodeficiency, Chemokine signaling pathway, and others. The immune related pathways with downregulated genes demonstrate that there is decreased immune activity in UHR-NB.

Upregulated immune-related genes in UHR-NB were enriched in P53 signaling pathway. The 26 commonly upregulated genes in UHR-NB are presented in the heatmap (Figure 6B). Gene set enrichment (GSE) analysis indicated that the 26 upregulated genes were only enriched in p53 signaling pathway (FDR = 0.03). We further constructed a protein–protein interaction (PPI) network with the upregulated genes (Figure 6C). Twenty out of 26 genes were on the PPI network and functioned together. Particularly, there were three genes on the KEGG p53 signaling pathway, including TP53, PMAIP1, and CCNB2 (blue diamond nodes, Figure 6C). Interestingly, three out of four NB-specific genes we discussed earlier were also on the network (yellow triangle nodes, Figure 6C), including GAL, TWIST1, and ADAM22. TWIST1 and GAL both directly connected with TP53, while ADAM22 interacted with TP53 though TWIST1. TP53 was the most frequently mutated gene in human cancers and the overexpression of TP53 was associated with poor prognosis in various cancers, including Esophageal [21], Colorectal [22], pancreatic, and triple negative breast cancer [23]. TP53 and related signaling pathway are associated with immune regulations and target many tumor immunosupression-associated genes [24]. Genetically, although the somatic mutations of TP53 were rare in primary NBs at diagnosis, a germline missense variant in TP53 was identified as a candidate pathogenic mutation associated with HR-NBs [25], suggesting the important role of P53 signaling pathway in UHR-NB subtype. However, although there is robust evidence for TP53 in cancers, the molecular functions and biological mechanisms of TP53 pathway in UHR-NB are still poorly understood. More experimental validation is required in future studies.

## 3. Discussion

HR-NB is a heterogeneous disease with intrinsic subtypes; however, HR-NB patients have been treated in a similar fashion without additional risk stratification. Stratifying HR-NB patients accurately into risk groups and identifying subtype-specific therapeutic targets is crucial for personalized treatment and individualized medicine. Moreover, immunotherapy represents a promising approach for HR-NB, because it has relatively low long-term and cumulative toxicity, which is particularly important for children. In this paper, we first identified 283 NB-specific immune-related genes through comparison the RNA-seq expression profiles of pediatric pan-cancers (ALL, AML, NB, and WT). Those immune-related genes were solely expressed in NB and they provided nice candidates for downstream analysis. Subsequently, we applied the unsupervised spectral co-clustering algorithm to the HR-NBs of two independent cohorts (TARGET and GSE49719) and discovered three clusters for each cohort independently. Interestingly, we consistently identified a UHR-NB subtype with significantly worst OS in two independent cohorts. Therefore, the original HR-NBs were stratified into two clinically meaningful subtypes (UHR-NB and HR-NB). The survival times in UHR-NB of the two cohorts were also quite similar, despite the data being collected independently from different platforms, suggesting that UHR-NB was a reproducible and clinically important subtype.

Within the 283 NB-spefic immune-related genes, we identified a panel of 39 immune-related genes (with four up and 35 down) that were differentiated in UHR-NB, as compared with HR-NB subtype. We validated our results in multiple independent cohorts. The 35 downregulated genes in UHR-NB were enriched in seven immune-related KEGG pathways, including Complement and coagulation cascades and Cell adhesion molecules (CAMs). The four UHR-NB upregulated genes were all overexpressed in MYCN amplified NB, and correlated with MYCN amplification in five additional independent cohorts. This finding was important, since some HR-NB associated oncogenes, such as MYCN, are difficult to target directly. Researchers struggle to crystallize MYC proteins for structural analyses, making it difficult to develop therapies that directly targeted the MYCN protein. NB-specific immune-related genes provide potential alternatives for therapeutic targets. Moreover, we demonstrated that two of the four upregulated genes (AMAM22 and TWIST1) were positively correlated with tumor stage in two independent cohorts, and KLHL13 was upregulated in subjects lacking of telomere maintenance, indicating the clinical relevance of the UHR-NB upregulated genes. Heterozygous deletions of 1p or 11q and gain of 17q were known genetic alterations in HR-NBs. We further showed that TWIST1 was associated with 1p and 11q deletion and 17q gain, and was upregulated in original HR-NBs. Finally, we investigated the prognostic effects of the four UHR-NB upregulated genes, and discovered that GAL was a highly significant OS predictor independent of age and MYCN amplification in two independent cohorts, while TWIST1 was statistically significant OS predictor in TARGET, after adjusting for age and MYCN, indicating their clinical implications.

To further investigate the biological characteristics of UHR-NB subtype, we identified 26 commonly upregulated and 311 downregulated genes in UHR-NB with all 4723 immune-related genes, as compared with HR-NB subtype in two independent cohorts. Eighty-one immune related pathways were enriched in the set of 311 downregulated genes, and only the P53 signaling pathway was enriched in the 26 upregulated genes, suggesting that the UHR-NB subtype was associated with depressed expression of immune-related genes and lower activities of immune-related pathways. PPI network analysis demonstrated that two NB-specific immune-related genes, GAL and TWIST1, directly connected to TP53, while ADAM22 interacted with TP53 through TWIST1, which suggested that UHR-NB was a P53 related subtype, although the molecular mechanisms of TP53 in UHR-NB were still poorly understood.

## 4. Materials and Methods

Our approach is involved in the integration of gene expression datasets of multi-cohorts extracted from public-domain, unsupervised biclustering, and bioinformatics analysis. The results are validated with multiple independent cohorts.

### 4.1. Datasets

#### 4.1.1. RNA-Seq Data for Pediatric Pan-Cancer from TARGET

The TARGET (https://ocg.cancer.gov/programs/target) database includes six different pediatric tumors with a total of 716 patient subjects available. RNA-seq transcriptome data with 58581 genes (transcripts) in log2 count for all subjects are pre-processed and ready for further analysis. Within the 716 pediatric tumors, there are 194 acute lymphoblastic leukemia (ALL), 224 acute myeloid leukemia (AML), two clear cell carcinoma of the kidney, 11 clear cell sarcoma of the kidney, 162 neuroblastoma (NB), and 123 wilms tumor (WT) subjects. In our analysis, we exclude the samples of two kidney tumors with small sample size, and concentrate our analysis on four types of tumors (ALL, AML, NB, and WT) with large sample sizes.

#### 4.1.2. Neuroblastoma Gene Expression Data from TARGET

The expression data from Affymetrix Human Exon Array platform has 247 samples (after excluding two samples with missing information). There are 217 high-risk and 30 low-risk NB patients and total 23434 genes available. Clinical metadata include age, gender, MYCN status, PLOIDY, stage, race, risk, overall and event free survival (OS and EFS), providing a nice source for studying the gene expression patterns of high-risk neuroblastoma. We will concentrate on 4723 genes that have immune system-related functions. The gene list was downloaded from InnateDB (https://www.innatedb.com/).

#### 4.1.3. GSE49710 RNA-Seq Data

The gene expression profiles downloaded from GEO (www.ncbi.nlm.nih.gov/geo/) have 498 primary neuroblastoma patient samples and 19,320 expressed genes [26]. Clinical metadata, including age, gender, stage, mycn status, risk status, overall, and event-free survival are also available. Again, we concentrate on studying 4723 immune related genes and 176 high-risk NB patients.

#### 4.1.4. Other Neuroblastoma Patient Datasets from GEO:

Several other NB datasets were used for exploring the associations and mechanisms of the immune-related genes with neuroblastoma, including

- GSE19274 [27]: it includes the expression data of neuroblastoma primary tumors with the Illumina Human 6 version 2 expression l bead chip. There are 100 samples and total 19,937 genes. Clinical information including age, gender, stage, MYCN and PLOIDY status, and risk categories is available, but there is no patient survival information in this data.
- GSE45547 [28]: the expression profiles from 649 neuroblastoma tumors were originally generated using 44K oligonucleotide microarrays. There are a total of 19,320 genes in the data, and clinical information including age, gender, stage, and mycn status, and survival are available, but there is no risk stratification in the data.
- GSE73517 [20]: the RNA expression profiles of 105 primary neuroblastomas were originally generated with the 4×44k oligonucleotide microarrays. There are 105 NB samples and 19,320 genes available. Clinical metadata include age, stage, risk, MYCN status, and heterozygous deletions of 1p or 11q and gain of 17q.
- GSE85047 [29]: this is the expression profiles of 283 primary untreated neuroblastoma tumors with Affymetrix Human Exon 1.0 ST Array. There are 13,489 expressed genes. All of the tumor samples are fully annotated including patient age at diagnosis, overall and progression free survival and MYCN amplification status.
- GSE120572 [6]: the gene expression profiles of 394 NB samples were originally produced with Agilent-020382 Human Custom Microarray 44k. There are 30,853 genes (transcripts) in the data. Clinical metadata, including age, stage, MYCN, and TERT status, are available.

### 4.2. Methods

Given a m×n expression matrix *X* with *m* rows of samples and *n* columns of genes, biclustering is an unsupervised machine learning technique that assigns both samples and genes into clusters simultaneously. Each resulting cluster is a submatrix of the original expression matrix that is based on some optimization criteria. There are many bi-clustering algorithms in the literature [30,31]. We utilize the spectral co-clustering algorithm originally proposed by Dhillon [32] to detect the subtypes of HR-NB. The idea of spectral co-clustering algorithm is based on bipartite graph model. The samples and genes are two sets of nodes, and each entry of matrix *X* corresponds to an edge between a patient sample and a gene. We first formulate a (m+n)×(m+n) (adjacency) graph matrix *A* from the original expression matrix with

$$A=\left[\begin{array}{cc} 0 & X\\ X^{T} & 0 \end{array}\right],\;\mathrm{then}\;\mathrm{the}\;\mathrm{Laplacian}\;\mathrm{matrix}:\;L=D-A,$$

where *D* is a diagonal matrix with $D_{ii}=\sum_{k}A_{ik}$, so $L_{ij}=\left\{\begin{array}{cc} \sum_{k}A_{ik}, & \mathrm{if}\;i=j,\\ -A_{ij}, & \mathrm{if}\;i\neq j. \end{array}\right.$

The approximating solution of optimal normalized-cut of a graph can be discovered by the generalized eigenvalue decomposition of the Laplacian matrix *L*. Each sample and a gene belongs to exactly one bi-cluster with this algorithm. Interestingly, this algorithm is equivalent to solve a singular value decomposition of the normalized expression matrix *X*, which is smaller than *A* and more efficient. More precisely, assuming that there are k biclusters, we first normalize matrix *X* into $X_{n}$ by setting

$$X_{n}=R^{-1/2}XC^{-1/2},\;\mathrm{where}\;R=\left[\begin{array}{cccc} \sum_{j}X_{1j} & 0 & \ldots & 0\\ 0 & \sum_{j}X_{2j} & \ldots & 0\\ \vdots & \vdots & \ddots & 0\\ 0 & 0 & \ldots & \sum_{j}X_{mj} \end{array}\right]$$

is a m×m diagonal matrix, and $C=\left[\begin{array}{cccc} \sum_{i}X_{i1} & 0 & \ldots & 0\\ 0 & \sum_{i}X_{i2} & \ldots & 0\\ \vdots & \vdots & \ddots & 0\\ 0 & 0 & \ldots & \sum_{i}X_{in} \end{array}\right]$ is a n×n diagonal matrix. We then find the singular value decomposition of $X_{n}$ by $X_{n}=U\Sigma V^{T}$. The left singular vectors *U* can be used to partition the samples, while the right singular vectors *V* are utilized for gene clustering.

With *k* bi-clusters, $p=\lceil \log_{2}k\rceil$ number of singular vectors are required for the bi-clustering. Excluding the trivial left and right eigenvector $u_{1}={\left[1,1,\ldots ,1\right]}^{T}$, and $v_{1}={\left[1,1,\ldots ,1\right]}^{T}$ with the largest singular value λ=1, U consists of $u_{2},u_{3},\ldots ,u_{p+1}$ singular vectors, and *V* contains the first $v_{2},v_{3},\ldots ,v_{p+1}$ singular vectors. They are used to form a matrix *Z*: $Z=\left[\begin{array}{c} R^{-1/2}U\\ C^{-1/2}V \end{array}\right]$. The standard k-means algorithm is then applied to the rows of *Z* for clustering. The first m-rows provide sample partitions, while the remaining n-rows in *Z* form gene clustering. The samples and genes can be rearranged to be contiguous to obtain the block diagonal or checkerboard bi-cluster structures.

Spectral co-clustering treats genes and samples as nodes of a connected graph and clusters are found by partitioning this graph, based on its spectral decomposition, into subgraphs. While there are many clustering methods in the literature (e.g., kmeans, hierarchical clustering), spectral co-clustering has its advantages. Gene expression data are usually noisy and have high-dimensions. Standard clustering methods based on original expression data do not perform well because of the curse of dimensionality. Spectral co-clustering method, on the other hand, projects the high-dimensional expression data onto low-dimensional space with singular value decomposition, and then detects the clusters based on the low-dimensional data. This approach reduces both dimensionality and noises of the data. Therefore, spectral co-clustering is more robust, which makes it a good technique for gene expression data clustering.

The software tools for analysis and visualization include the statistical and machine learning toolbox in MATLAB R2109a (https://www.mathworks.com/), VennDiagram [33], Clusterprofiler [34], and survminer packages (https://github.com/kassambara/survminer) in R, and cytoscape 3.7.1 (https://cytoscape.org/) [35]. Statistical tests such as Student’s *t*-test, and log-rank test for Kaplan Meier curves are also used in our analysis. Gene enrichment analysis is performed by STRING (https://string-db.org/) with the minimum required interaction score of 0.3 and false discovery rate (FDR) of 0.05.

## 5. Conclusions

In summary, we conclude that the expression patterns of 283 immune-related genes successfully stratify original HR-NB patients into two clinically distinct subtypes (UHR-NB and HRNB). UHR-NB is a clinically important subtype with the worst overall survival. This subtype was robust and reproducible in multiple independent cohorts. NB-specific upregulated immune-related genes, including GAL, TWIST1, ADAM22, and KLHL13, might have important clinical implications for UHR-NBs.

## Supplementary Materials

The following are available at https://www.mdpi.com/2072-6694/12/7/1739/s1, Figure S1: Detailed Kaplan-Meier survival curves for both TARGET and GSE49710. The overall survival (OS) of ultra-high risk neuroblastoma (UHR-NB) vs high risk neuroblastoma (HR-NB). A, OS in TARGET. B, the OS for GSE49710, Table S1: The average expression of 283 NB-specific immune-related genes in 4 pediatric tumors. The gene expression was normalized with log2 transformation and quantile normalization, Table S2: Three Subtypes in TARGET and GSE49710 cohorts. The subtypes were discovered with spectral co-clustering algorithm, Table S3: Differentiated NB-specific immune-related genes in both cohorts, when comparing UHR-NB with HR-NB. The genes were selected from 283 NB-specific immune-related genes with more than 1.5 fold changes and the *p* value of less than 1.0 $\times 10^{-3}$, Table S4: Overall survival for 39 NB-specific differential genes with Cox regression in two cohorts, where Beta is the estimated coefficient for each gene with univariate and multivariate Cox regression, Hazard Ratio denotes the marginal hazard ratio for each gene, HR_LB is the 95% lower bound of hazard ratio, and HR_UB is the 95% upper bound of the hazard ratio, Table S5: Differentiated immune-related genes in both cohorts, when comparing UHR-NB vs HR-NB. The genes were selected with the 4723 immune-related genes from InnateDB (https://www.innatedb.com/). More than 1.5 fold and $p<1.0\times 10^{-3}$ were used as a cutoff for gene selection, Table S6: KEGG pathways for 311 downregulated immune-related genes in UHR-NB subtype. 43 KEGG pathways with molecular/metabolic/signialing functions were enriched in HR-NB with FDR<0.05.

## Abbreviations

The following abbreviations are used in this manuscript:

HR-NB	high risk neuroblastoma
UHR-NB	ultra-high risk neuroblastoma
NB	neuroblastoma
ALL	acute lymphoblastic leukemia
AML	acute myeloid leukemia
WT	Wilms tumor
TARGET	tumor alterations relevant for genomics-driven therapy
ADAM22	A Disintegrin And Metalloproteinase Domain 22
GAL	Galanin And GMAP Prepropeptide
KLHL13	Kelch Like Family Member 13
TWIST1	Twist Family BHLH Transcription Factor 1
OS	overall survival
EFS	event-free survival
MYCN	MYCN Proto-Oncogene, BHLH Transcription Factor.
